# Leukocyte Beta-Catenin Expression Is Disturbed in Systemic Lupus Erythematosus

**DOI:** 10.1371/journal.pone.0161682

**Published:** 2016-08-22

**Authors:** Jacob J. Orme, Yong Du, Kamala Vanarsa, Tianfu Wu, Anne B. Satterthwaite, Chandra Mohan

**Affiliations:** 1 The Department of Internal Medicine Rheumatic Diseases Division, University of Texas Southwestern Medical Center, Dallas, Texas, United States of America; 2 The Department of Biomedical Engineering, University of Houston, Houston, Texas, United States of America; National Cancer Center, JAPAN

## Abstract

Wnt/β-catenin signaling is relatively understudied in immunity and autoimmunity. β-catenin blocks inflammatory mediators and favors tolerogenic dendritic cell (DC) phenotypes. We show here that leukocytes from lupus-prone mice and SLE patients express diminished β-catenin transcriptional activity, particularly in myeloid cells, although other leukocytes revealed similar trends. Serum levels of DKK-1, an inhibitor under transcriptional control of Wnt/β-catenin, were also decreased in lupus-prone mice. Surprisingly, however, preemptive deletion of β-catenin from macrophages appears to have no effect on lupus development, even in mice with varying genetic loads for lupus. Although myeloid-specific loss of β-catenin does not seem to be important for lupus development, the potential role of this transcription factor in other leukocytes and renal cells remain to be elucidated.

## Introduction

The Wnt/β-catenin or “canonical” Wnt pathway is a ubiquitous, conserved signaling pathway. First characterized in *Drosophila* as homolog Armadillo, β-catenin functions in cell adhesion as well as in downstream control of transcription [1–3]. β-catenin transcriptional activity is the result of canonical Wnt signaling (see S1 Fig). In brief, soluble Wnt engages the seven membrane-spanning receptor Frizzled, activating Disheveled (Dsh). Disheveled blocks the beta catenin destruction complex, comprised of Axin, APC, and GSK-3. In the absence of Wnt signaling, this complex binds and phosphorylates β-catenin at Serine 33, Serine 37, and Threonine 41, leading to ubiquitination and degradation [4, 5]. In the presence of Wnt signaling Dsh inhibits this complex, allowing β-catenin to translocate to the nucleus and transcribe such targets as Axin-2, matrix metalloprotease 7, PPARδ, CD44, cyclin D1, and c-Jun [6].

This transcriptional activity is involved in multiple cell fate decisions in the embryo and adulthood. Armadillo/β-catenin knockout leads to gross malformation in *Drosophila* and embryonic lethality in mice [7, 8]. Breakdown in β-catenin regulation due to APC mutations in human patients (termed Familial Adenomatous Polyposis or FAP) leads to sporadic colorectal cancers [9, 10]. Aberrations in other Wnt/β-catenin pathway factors also contribute to Wilm’s tumor and hepatocellular carcinoma (HCC), among others [5, 11–13].

The Wnt/β-catenin pathway also plays roles in immunity, contributing to hematopoietic stem cell (HSC) maintenance, thymocyte differentiation, pre-B cell proliferation, and the type I interferon response [14–17] In macrophages, β-catenin blocks the inflammatory transcription factor NFκB [18, 19]. Previous work has shown that β-catenin is tolerogenic in several autoimmune models such as DSS-induced IBD, EAE, and rheumatoid arthritis [20–23]. These prior studies have focused mainly on the role of β-catenin in dendritic cells and regulatory T cells.

We and others have screened lupus-prone mice and SLE patient serum and find increased β-catenin-related markers [24]. Given the interesting role of this pathway in immunity and tolerance, and the observed changes in several circulating proteins related to this signaling pathway, we examined the potential role of this pathway in lupus. Specifically, the role of leukocyte β-catenin in lupus has not been explored. In the present work we determine that β-catenin expression is lost from the spleens of lupus-prone mice in a transcription-dependent manner. We demonstrate that human SLE PBMCs express less β-catenin than healthy PBMCs. We show that a macrophage-specific knockout of β-catenin does not further affect disease progression in the B6.Sle1 or B6.Sle1.Yaa lupus-prone mouse model. Finally, we discuss potential roles for β-catenin in SLE.

## Materials and Methods

### Animal Subjects

Congenic control (C57BL/6 or B6), Mrl-lpr, Ctnnbfl/fl [25], and Lyz-M-Cre [26] mice were purchased from the Jackson Laboratory (Bar Harbor, ME) or Taconic Farms (Hudson, NY). B6.Sle1 [27], B6.Sle3 [28], B6.Yaa [29], and BWF1 [30] mice were bred in our mouse colony. Mice used for this study were 3 to 13 month females or—in the case of mice harboring the Yaa locus and their controls—males maintained in a specific pathogen-free environment. Mice were euthanized using a CO2 chamber and cervical dislocation. The Institutional Animal Care and Use Committee at the University of Texas Southwestern Medical Center approved all experiments on mice.

### Human Subjects

Patient samples were collected from consecutively seen patients at a Rheumatology outpatient clinic at UT Southwestern Medical Center, without any pre-selection. All patients fulfilled ACR classification criteria for the diagnosis of SLE. Patient demographics are summarized in S1 Table. Collection of peripheral blood from consented human subjects was overseen and approved by the University of Texas Southwestern Medical Center Institutional Review Board (IRB). Controls were matched where possible by age, gender, and ethnicity (see S1 Table).

### Western Analysis

Splenocytes or PBMCs were isolated by centrifugation alone or on a Ficoll gradient, lysed, and prepared for Western Blot analysis in sample buffer by boiling for 10 minutes. Samples were spun down, subjected to SDS-PAGE, and transferred using standard procedures. Blots were probed for β-catenin (Pierce, Rockford, IL), pSer33 β-catenin (Pierce, Rockford, Il.), Axin-2 (Abcam, Cambridge, MA), GAPDH (Cell Signal, Beverly, MA), and β-actin (Santa Cruz, Santa Cruz). Secondary HRP-conjugated antibodies (GE, Fairfield, CT) were selected against primary antibody epitopes as appropriate. For analysis, bands were quantified using ImageJ®.

### Immunohistochemistry (IHC) and Immunofluorescence (IF)

Kidneys and spleens were isolated and laterally hemisected and either fixed in OCT and frozen for immunofluorescence (IF) or embedded in paraffin for immunohistochemistry (IHC). Sections comprised 3-5mm and antigens were exposed with a sodium citrate buffer. Sections were blocked in 5% milk prior to staining with rabbit anti-β-catenin (Pierce, Rockford, IL), rabbit anti-pSer33 β-catenin (Pierce, Rockford, Il.), and APC-conjugated anti-F4/80 (R&D Systems, Minneapolis, MN). Anti-Rabbit HRP conjugate antibodies were used as a secondary stain (Life Technologies, Carlsbad, CA)

### Reverse Transcriptase Polymerase Chain Reaction (RT-PCR)

RNA was extracted from tissue and reverse-transcribed to produce cDNA using standard protocols. In brief, splenocytes were harvested, filtered through a fine mesh, depleted of red blood cells by hypotonic solution, and pelleted. Alternatively, PBMCs were harvested by centrifugation over a Ficoll gradient and isolated from the buffy coat layer. Each sample was then resuspended and sonicated in 1ml TRIzol® Reagent (Life Technologies #15596–026) and left at room temperature for 5 minutes. 200μl chloroform was then added to each sample and each sample was vortexed 15 seconds and left at room temperature for 3 minutes. Samples were then centrifuged at 12,000 x g for 15 minutes and the top (aqueous) layer was transferred to a new tube. 500μl isopropanol were added to each tube and left at room temperature for 10 minutes. Samples were then centrifuged at 12,000 x g for 10 minutes and supernatant was aspirated. Pellets were washed with 500μl 80% ethanol (w/v) and centrifuged at 7,500 x g for 5 minutes. Supernatant was removed carefully by pipette. Samples were left to air dry 2–3 minutes followed by incubation at 70°C for 2–3 minutes. Resultant RNA was resuspended in 80μl and quantified using NanoDrop® 1000 (Thermo Fisher).

cDNA was produced from the foregoing RNA using a High Capacity cDNA Kit (Applied Biosystems, Foster City, CA). In brief, a master mix was prepared with 2μl 10X RT-PCR buffer, 2μl10x RT primers, 0.8μl 100μM dNTP mix, and 1μl transcriptase per sample. Water and isolated RNA were added to 14.2μl and 1μg RNA for a final volume of 20μl in each sample. Samples were treated with thermocycler at 25°C for 10 minutes, 37°C for 120 minutes, and 85°C for 5 minutes.

The resultant cDNA was then detected using Q-PCR with SybrGreen® using the following probes: Cyclophilin A for 5’-cattatggcgtgtaaagtcacc-3’, Cyclophilin A rev 5’- gcagacaaagttccaaagacag-3', Ctnnb for 5’- aaaatggcagtgcgtttag -3', Ctnnb rev 5’- tttgaaggcagtctgtcgta -3', Axin2 for 5’- ctggctccagaagatcacaa -3’, Axin2 rev 5’- aggtgacaaccagctcactg -3’, MMP7 for 5’- gtatggggaactgctgacatcatg -3’, MMP7 rev 5’- ctgaatgcctttaatatcatcctg -3’. Each sample was normalized to Cyclophilin A control using ΔCt to compute relative expression.

### Enzyme-Linked Immunosorbance Assays

Anti-dsDNA antibodies were detected using dsDNA-coated plates and alkaline phosphatase-conjugated anti-IgG antibodies (Southern Biotech, Birmingham, AL). In brief, wells are pre-coated with mBSA, coated with dsDNA from calf thymus, blocked with 3% BSA and 3mM EDTA in 0.1% gelatin PBS, probed with sera, and analyzed using HRP-conjugated anti-mouse IgG or IgM antibodies or by colorimetric dye. Serum creatinine (Cayman, Ann Arbor, MI) was detected by ELISA per the manufacturers’ protocols.

### Flow Cytometry

Cells were isolated from spleens as above, washed with PBS plus 1% BSA, and stained intracellularly with A555-conjugated anti-β-catenin antibody (Pierce, Rockford, Il.) or A555-conjugated isotype control using methanol intracellular staining buffer. Cells were further stained with cell-specific markers for CD11b, CD19, CD4, and CD8. Data were analyzed using FlowJo® (Treestar. Stanford) flow cytometry software.

### Enzyme-Linked Immunosorbent Assay (ELISA)

ELISA quantifying serum DKK-1 and sFRP were performed using kits from R&D (Minneapolis, MN) and MyBioSource (San Diego, CA), respectively. In brief, sera were prepared from individual mice by centrifugation of whole blood for 20 minutes at 4 C. Diluted samples and standards were added to antibody-coated 96-well plates, washed per protocol, and assayed using conjugated HRP with TMB substrate, and detected by absorbance at 460nm.

### Statistical Procedures

P values where not otherwise noted were determined using a student’s t test using GraphPad® Prism software. Significance cutoffs for p values were set at 0.05. For Western Blot analysis, bands were quantified using ImageJ®.

## Results

### Lupus Prone Mouse Splenocytes and Human SLE PBMCs Exhibit Diminished β-Catenin Levels

**Fig 1:**
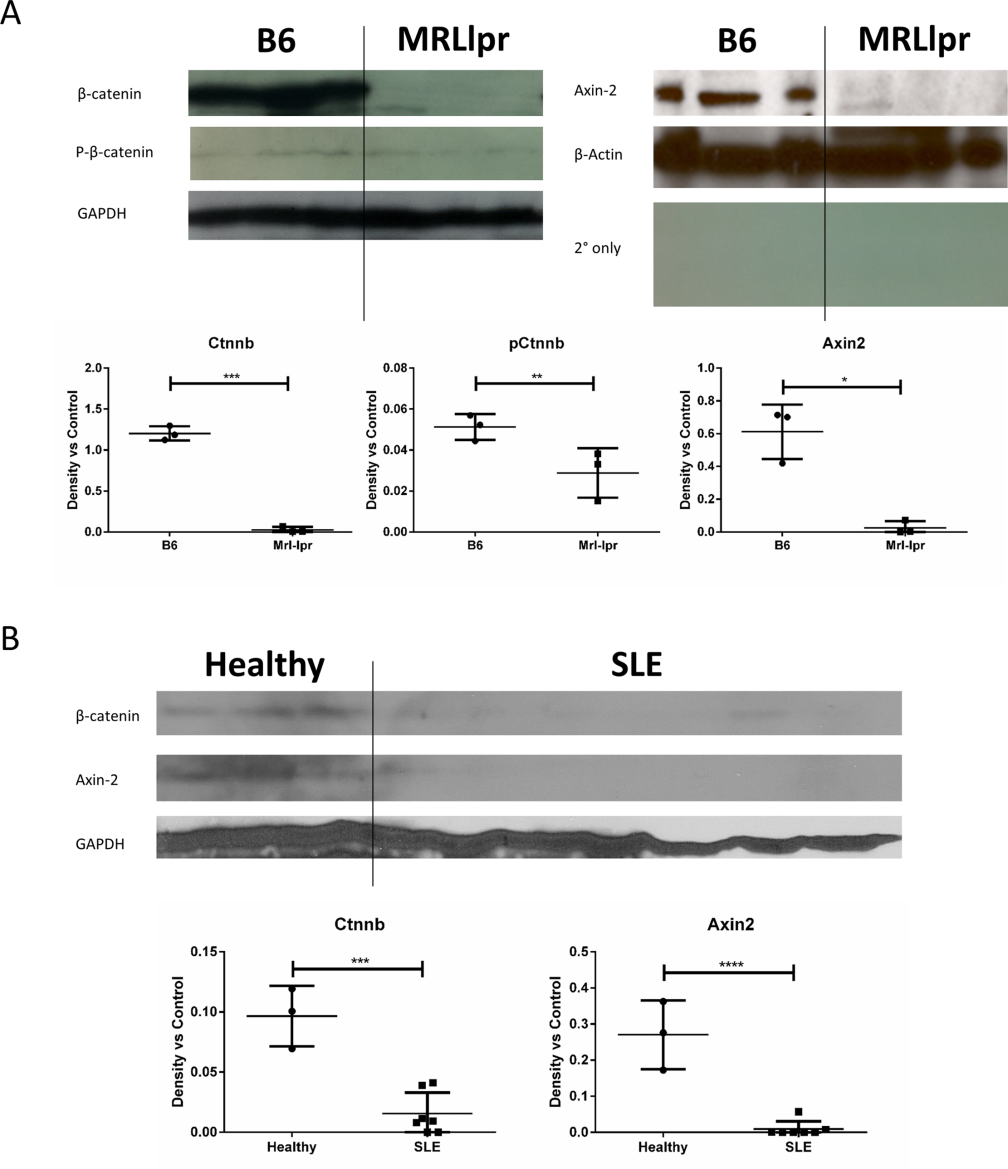
B-catenin and its targets are decreased in lupus-prone Mrl-lpr mouse and human SLE splenocytes. (A) Splenocytes from 6-month-old healthy B6 control and lupus-prone Mrl-lpr mice were analyzed by Western blot for total β-catenin, phosphorylation-inactivated (pSer33) β-catenin, negative feedback target Axin-2, and loading controls. Mrl-lpr splenocytes exhibit a loss of β-catenin (p = 0.0011) and Axin-2 (p = 0.0015) as quantified by densitometry using ImageJ. (B) Peripheral blood mononuclear cells (PBMCs) from SLE patients and healthy controls were analyzed by Western blot for total β-catenin, negative feedback target Axin-2, and loading controls. SLE PBMCs exhibit a loss of β-catenin (p = 0.0003) and Axin-2 (p<0.0001) as quantified by densitometry using ImageJ. All plotted data have been normalized against the GAPDH loading control.

To determine whether leukocyte β-catenin activity may contribute to serum β-catenin markers we observed in lupus, we isolated splenocytes from age-matched healthy B6 control and lupus-prone Mrl-lpr mice. We analyzed cell lysates by Western blot and probed for total β-catenin, phosphorylation-inactivated (pSer33) β-catenin, β-catenin transcription target Axin-2, and loading controls (Fig 1A). Lupus-prone Mrl-lpr splenocytes demonstrated a dramatic reduction in both β-catenin and Axin-2, a transcriptional target of β-catenin. There was a greater decrease in total relative to pSer33 β-catenin, suggesting that any residual β-catenin protein in the Mrl.lpr splenocytes was inactive.

To determine whether leukocyte β-catenin activity is altered in patients with SLE, we isolated peripheral blood mononuclear cells (PBMCs) from healthy controls and SLE patients. We analyzed cell lysates by Western blot and probed for total β-catenin, β-catenin transcription target Axin-2, and loading controls (Fig 1B). SLE PBMCs lacked β-catenin and the β-catenin transcriptional target, Axin-2.

### Lupus Prone Mouse Splenocytes Exhibit a Loss of Beta Catenin Transcriptional Activity

**Fig 2:**
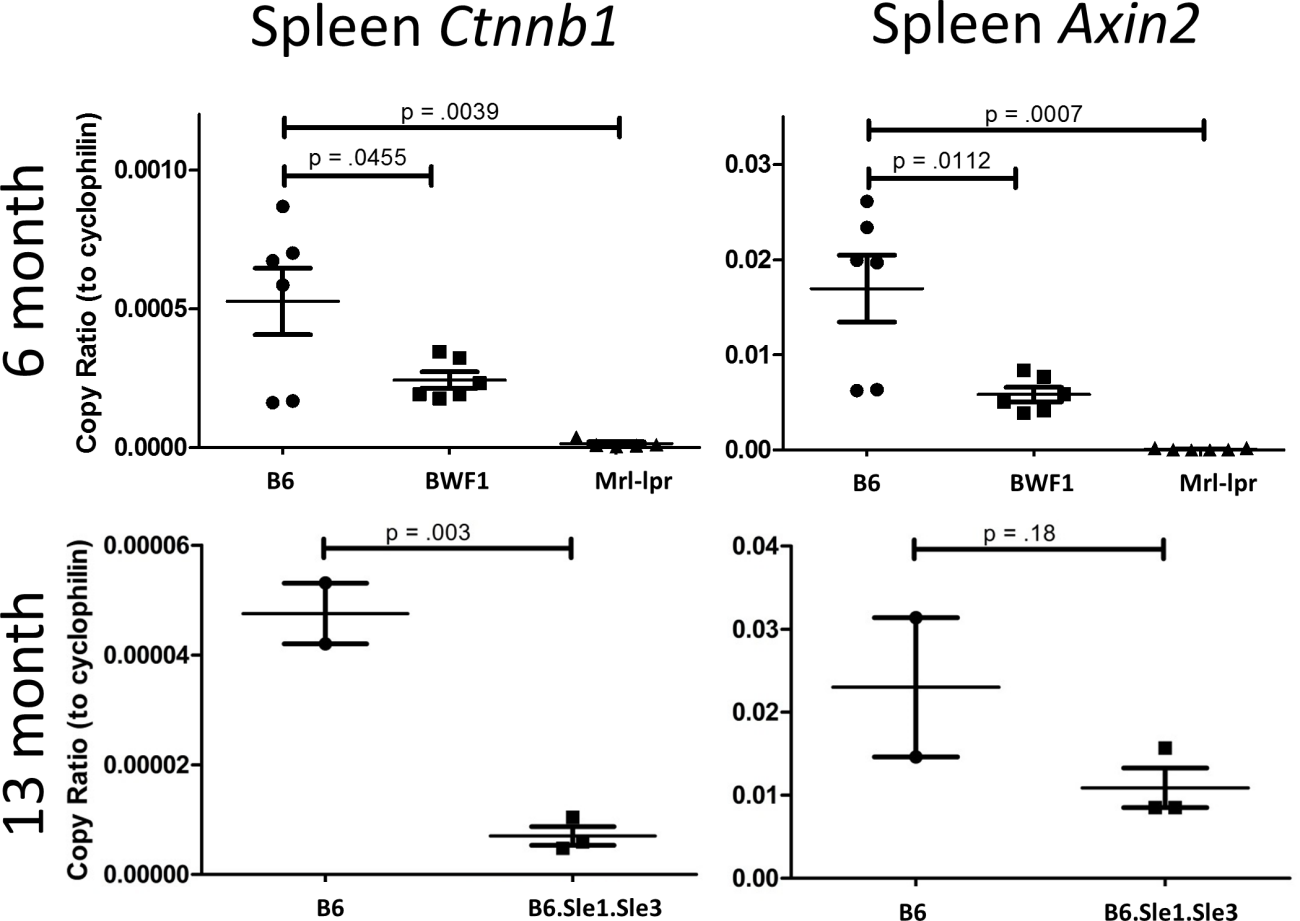
Splenocytes from multiple, genetically diverse lupus-prone strains express lower levels of β-catenin target transcripts. Splenocytes from 6-month and 13-month healthy B6 and lupus-prone Mrl-lpr, BWF1, and B6.Sle1.Sle3 mice were isolated and probed for *Ctnnb1* and *Axin2* transcripts by RT-PCR. Lupus-prone mice exhibit a loss of β-catenin-related transcription versus healthy age-matched B6 controls.

**Fig 3:**
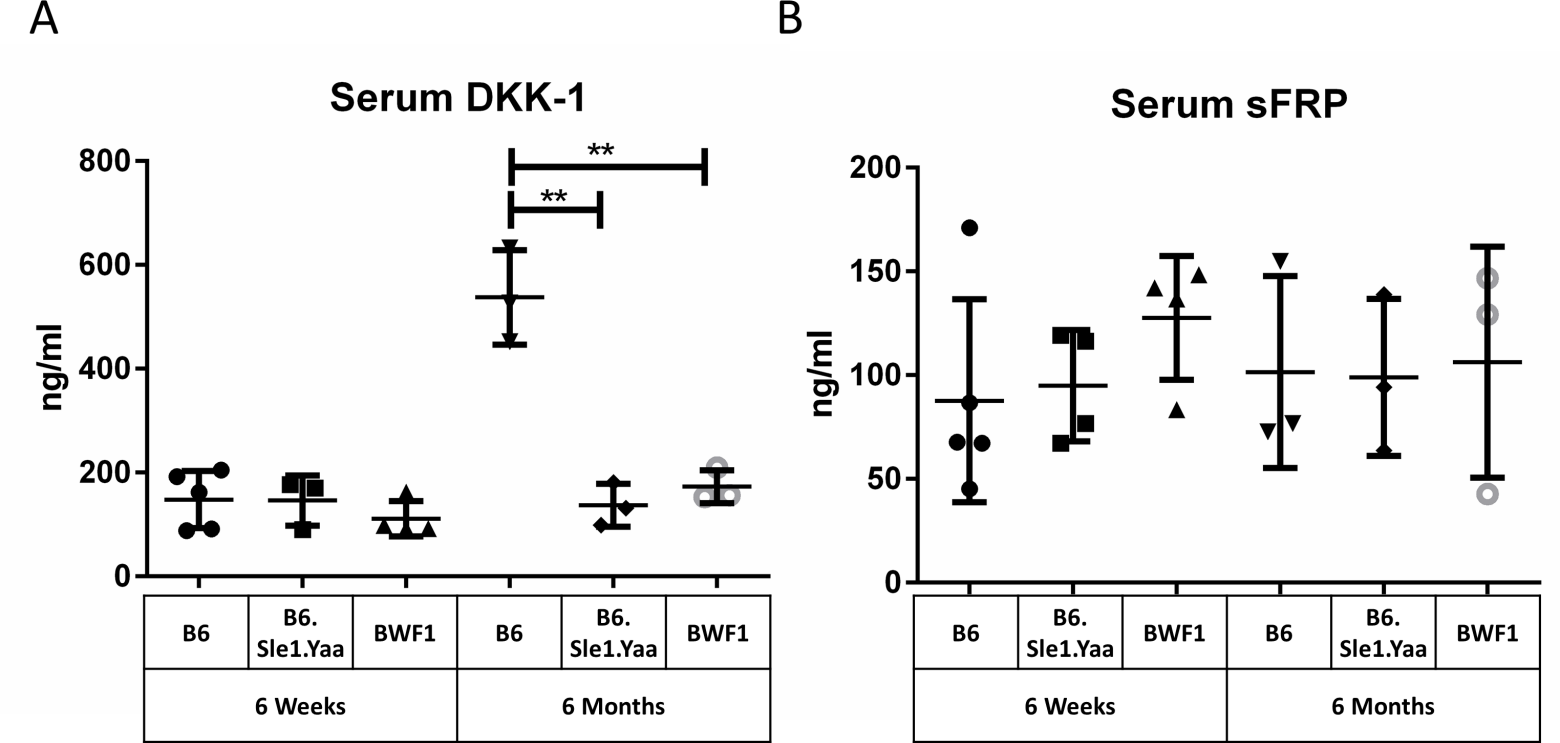
Serum DKK-1 is suppressed in lupus-prone mice over time. Sera from young (6 week) and aged (6 month) B6 healthy control, B6.Sle1.Yaa lupus-prone, and BWF1 lupus-prone mice were collected and assayed by ELISA for DKK-1 and sFRP. (A) DKK-1, an inhibitor under transcriptional control of Wnt/β-catenin, did not rise over time in lupus-prone mice. (B) Serum sFRP, which by contrast is an epithelial cell-restricted Wnt/β-catenin transcription target, showed no such changes.

To characterize the transcriptional activity of β-catenin in whole splenocytes, we isolated RNA from splenocytes from age-matched healthy B6 control and lupus-prone Mrl-lpr, BWF1, and B6.Sle1.Sle3 mice. Lupus-prone mouse splenocytes express diminished *Ctnnb1* (the gene encoding β-catenin) and *Axin2* mRNA (Fig 2). Loss of β-catenin transcriptional activity is consistent with reduced protein levels observed in Mrl-lpr splenocytes in Fig 1. Serum levels of DKK-1, an inhibitor under transcriptional control of Wnt/β-catenin, did not rise over time in lupus-prone mice as they did in healthy B6 mice (Fig 3A). Serum sFRP, which by contrast is an epithelial cell-restricted Wnt/β-catenin transcription target, showed no such changes (Fig 3B).

**Fig 4:**
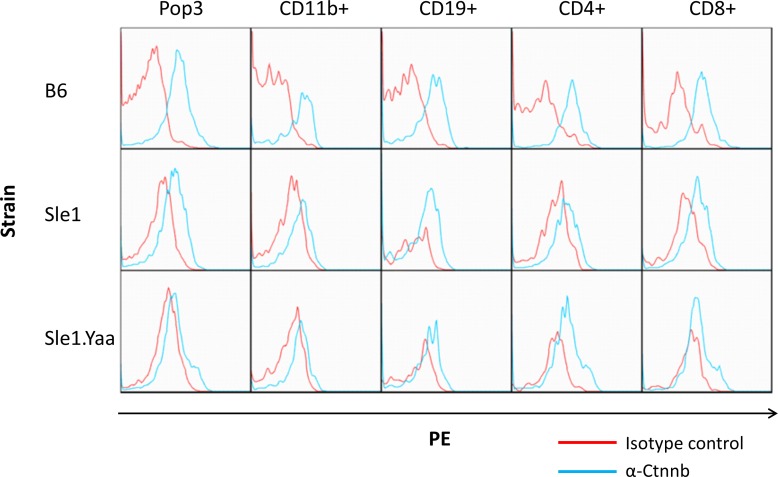
Lupus-prone CD11b+ splenocytes express reduced β-catenin. Splenocytes were extracted from 8-month-old healthy B6, lupus-prone B6.Sle1, and lupus-prone B6.Sle1.Yaa mice and analyzed by flow cytometry for intracellular β-catenin (blue) versus isotype control staining (red). Representative plots shown. Pop3 represents total live cells. CD11b+ cell β-catenin was significantly lower in lupus-prone splenocytes versus healthy controls (by MFI ratios, p = 0.0476).

To determine the leukocyte subset expression pattern of β-catenin and to determine whether an additional lupus-prone strain also lacked leukocyte β-catenin expression, we isolated splenocytes from healthy B6 and lupus-prone B6.Sle1 and B6.Sle1.Yaa mice and stained them intracellularly with anti-β-catenin or isotype control antibody and analyzed by flow cytometry. A representative plot for each strain is shown in Fig 4. While all healthy splenocytes expressed some level of β-catenin, lupus-prone B6.Sle1 and B6.Sle1.Yaa splenocytes appear to have lost β-catenin. This loss of β-catenin was most significant in macrophages and more pronounced in the more-diseased B6.Sle1.Yaa samples, though similar trends were also noted in B-cells and T-cells.

### LyzM-Cre-Driven Loss of Beta Catenin Does Not Affect Lupus Development in B6.Sle1 and B6.Sle1.Yaa Mice

**Fig 5:**
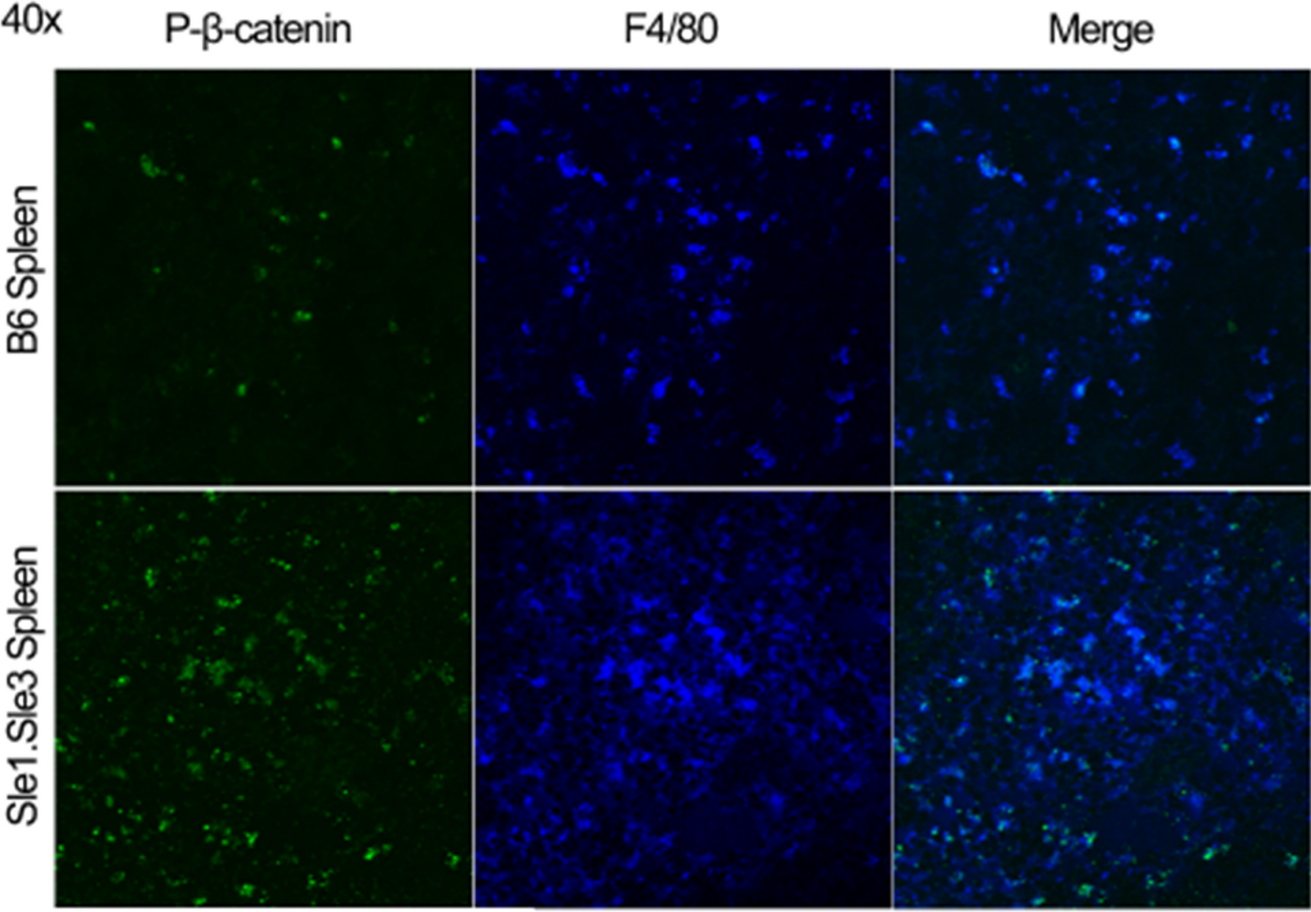
Lupus-prone F4/80-positive splenocytes express reduced β-catenin signaling. Spleens from 4 month old healthy B6 and lupus-prone B6.Sle1.Sle3 mice were sectioned and stained for phosphorylated (*i*.*e*. inactivated) β-catenin protein (green) and F4/80 (blue). These inactivated β-catenin proteins co-localized with F4/80-positive splenocytes (*i*.*e*. macrophages).

Β-catenin suppresses NFκB in normal macrophages [18, 19]. Macrophage NFκB induces many inflammatory mediators of lupus pathogenesis [31, 32]. Immunofluorescent staining of lupus-prone mouse spleens showed phosphorylated β-catenin in macrophages (Fig 5), consistent with remaining β-catenin being inactive in these cells. We hypothesized that the loss of β-catenin-mediated suppression of NFκB and related inflammatory factors in macrophages may contribute to lupus pathogenesis. We decided to test this hypothesis in mice that bearly develop lupus, B6.Sle1, as well as in mice that develop full-blown lupus, B6.Sle1.Yaa.

**Fig 6:**
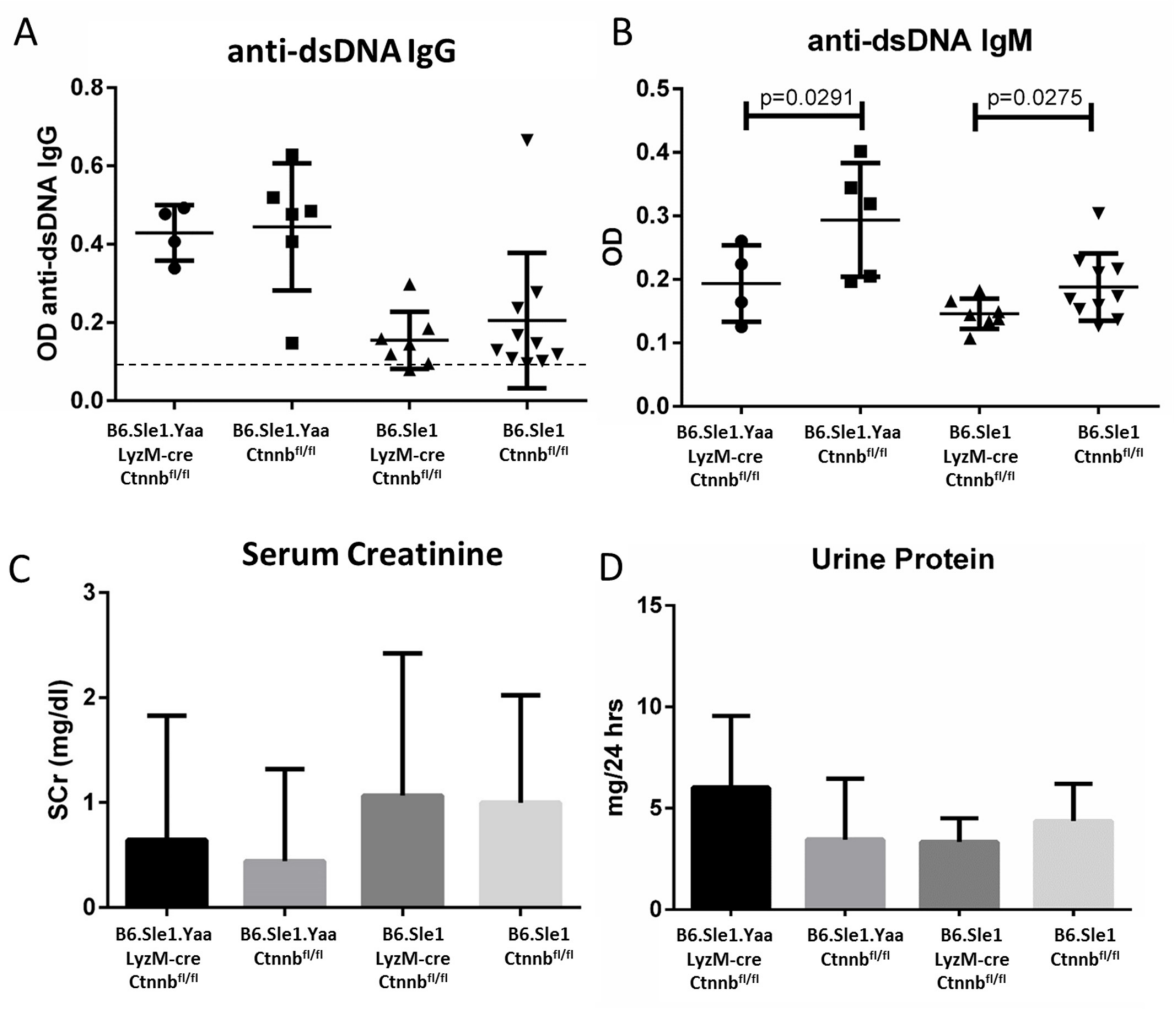
LyzM-cre-mediated β-catenin knockout does not affect lupus pathogenesis. B6 mice were bred to produce mice expressing the lupus-susceptibility locus *Sle1*, the lupus-accelerating locus *Yaa* (males only), and β-catenin-floxed loci in the presence or absence of the LyzM-cre locus. Mice expressing all of these loci are genetically lupus-prone and lack macrophage β-catenin expression. Mice expressing all but the LyzM-cre locus are sibling controls. Mice lacking macrophage β-catenin exhibit similar levels of anti-dsDNA IgG (A) but significantly lower levels of anti-dsDNA IgM (B). These mice did not exhibit significantly different markers of kidney function as measured by serum creatinine (C) and urine protein (D).

Thus, we bred LyzM-cre and floxed β-catenin (Ctnnb^fl/fl^) loci onto lupus-prone male B6.Sle1.Yaa and control B6.Sle1 mice and confirmed knockdown of expression by RT-PCR (S5 Fig). All offspring were aged and phenotyped for evidence of lupus. These mice did not exhibit significant changes in anti-dsDNA IgG, serum creatinine, or urine protein versus sibling controls (Fig 6). They also did not upregulate serum markers of the NFκB pathway such as IL-6. This was also true when examined at earlier time-points (data not shown). They did, however, exhibit significant reduction in anti-dsDNA IgM. These results suggest that early β-catenin loss in myeloid cells does not accelerate disease development, even in the context of a lupus-prone genetic background.

## Discussion

### Beta Catenin Loss and Its Potential Effects in SLE

In the present study, we found that LyzM-Cre-mediated macrophage-specific β-catenin knockout does not significantly accelerate disease in B6.Sle1 or B6.Sle1.Yaa lupus-prone models. This was surprising given that beta catenin is a known regulator of NFκB, and macrophage NFκB is important in lupus pathogenesis [19, 33]. Further, previous work has shown that monocytes are increased and activated in SLE, which would have favored an effect of β-catenin loss [34].

We considered several potential explanations for these observations. First, β-catenin may be lost relatively early in lupus pathogenesis. If this transcription factor is already missing from lupus-prone mice from very early, additional genetic removal would be unlikely to impact disease pathogenesis any further. However, his seems less likely given that serum DKK-1 inversely correlates with disease and because β-catenin loss is most pronounced in more advanced disease models [35] (Figs 2–4). Future studies using a floxed β-catenin *overexpression* allele may parse out this effect. Second, NFκB in lupus-prone macrophages may be driven by processes that are independent of physiologic β-catenin transcriptional control. In this case, loss of β-catenin may have no effect because the disease process has already overwhelmed β-catenin-mediated suppression of NFκB. These data would suggest that previously-proposed β-catenin inhibitors would not effectively alter macrophage phenotypes in SLE. Third, the null hypothesis also merits consideration—although β-catenin is lost from myeloid cells, it plays no active role in driving pathogenesis, and it may simply be a consequence of the disease.

We also have to entertain the possibility that the loss of β-catenin in other leukocytes may contribute to SLE pathogenesis. DCs, T cells, and B cells lose β-catenin in lupus (Fig 4). B cell NFκB overexpression alone is sufficient to induce lupus-like disease in mice [36]. The loss of β-catenin in T cells and dendritic cells worsens autoimmune models like IBD, EAE, and RA [20–23]. These findings suggest similar roles in SLE. For instance, β-catenin loss has previously been shown to diminish T cell anergy as well as regulatory T cell survival, either of which could contribute to SLE pathogenesis [37, 38]. These potential roles will require further study.

### Potential Causes of Leukocyte β-Catenin Loss in SLE

What causes the loss of β-catenin transcriptional activity in leukocytes? Wnt ligand is ubiquitously expressed in both healthy and SLE microenvironments and all leukocyte populations exhibit downregulation, suggesting that soluble factors downregulate β-catenin. DKK-1 is a soluble inhibitor of the Wnt/β-catenin pathway through Frizzled, whose expression is induced as a negative feedback mechanism in response to β-catenin. It is depressed in aged lupus-prone mouse serum relative to healthy controls, likely due to reduced activity of the β-catenin pathway. DKK-1 levels in human SLE correlate most closely with bone erosion and nephritis, perhaps indicating an autoregulatory feedback loop during disease progression. Other soluble inhibitors of Wnt/β-catenin signaling like CK1 and sFRP have not been reported in SLE. We found no change in sFRP in our models. We and others have reported elevated Interferon alpha in SLE serum, and SLE leukocytes display an “interferon signature” indicative of exposure to type I interferons. Furthermore, alpha interferon downregulates β-catenin transcription in cell lines [39, 40]. Thus increased type I interferon may cause β-catenin downregulation in leukocytes from SLE patients and in mouse models. These correlations are complex and will require further study.

### Beta Catenin Activity Is Increased in the Kidneys of Lupus-Prone Mice

Tveita and Rekvig previously showed that β-catenin-related transcription is elevated in the kidneys of lupus-prone BWF1 mice [24]. We have also observed that renal beta catenin expression and its transcriptional targets is elevated in another mouse model of lupus, B6.Sle1.Sle3 (S3 Fig). We further noted in our immunohistochemistry and immunofluorescence studies that β-catenin may localize near the surface of tubular epithelial cells in lupus-prone Mrl-lpr and BWF1 kidneys (S4 Fig). This observation, if confirmed, would be interesting because it is known that beta catenin operates not only as a transcription factor but also as an essential component of adherens junctions [41]. This has led to speculation about the potential roles of β-catenin in adhesion and transcription [42]. Adherens junctions are important in epithelial cell-cell adhesion and may increase in response to increased stresses at these junctions. Further study to confirm and explain these findings is warranted.

### Conclusion

In the foregoing work, we have determined that leukocytes from lupus-prone mice and SLE patients express diminished β-catenin transcriptional activity, particularly in myeloid cells, although other leukocytes revealed similar trends. Surprisingly, however, preemptive deletion of β-catenin from macrophages appears to have no effect on lupus development, even in mice with varying genetic loads for lupus. Although myeloid-specific loss of β-catenin does not seem to be important for lupus development, the potential role of this transcription factor in other leukocytes and renal cells remain to be elucidated.

## Supporting Information

S1 Fig The Wnt/β-Ctnnb pathway. (TIF)

S2 Fig Splenic Ctnnb-related transcription in age-matched B6 vs B6.Sle1.Yaa mice. Splenocytes from age-matched young (6 week) and old (6 month) healthy control B6 and lupus-prone B6.Sle1.Yaa mice were isolated and tested for *Ctnnb* and *Axin2* expression by RT-PCR. Older lupus-prone mice exhibited significantly lower *Ctnnb* and *Axin2* transcripts than healthy age-matched controls (p = 0.0354, p = 0.0259). In contrast, young lupus-prone mice expressed *Ctnnb* and *Axin2* transcripts at levels similar to those in young B6 controls.
(TIF)

S3 Fig Renal Ctnnb-related transcription in age-matched B6 vs B6.Sle1.Sle3 mice. Beta catenin expression and that of its transcriptional targets *Axin2* and *MMP7* were compared by RT-PCR in 4 month-old B6 healthy control and B6.Sle1.Sle3 lupus-prone kidneys. Transcription of these genes is elevated in B6.Sle1.Sle3 lupus-prone mice.
(TIF)

S4 Fig IF and IHC Ctnnb staining in healthy B6 vs BWF1 kidneys. (A) Kidneys were isolated, prepared, and stained for β-catenin (red) and macrophage marker F4/80 (blue). (B) Kidneys were isolated, prepared, and stained for total β-catenin (top) and inactivated/structural pSer33 β-catenin (bottom).
(TIF)

S5 Fig Knockdown of *Ctnnb* expression in peripheral leukocytes of B6.Sle1.Yaa. Peripheral blood was taken from young (2–4 wk) B6.Sle1.Yaa.Ctnnbfl/fl and B6.Sle1.Yaa.LyzM-cre mice, RNA was isolated, and RT-PCR was performed to determine gross levels of leukocyte *Ctnnb* in each mouse. Due to animal size, there was insufficient blood extracted for a subset of samples to perform RT-PCR.
(TIF)

S1 Table Patient Demographic Data. Gender, race, age, and SLEDAI scores are given for healthy controls and SLE patients from whom samples were obtained for Fig 1. Of SLE patients, four used Prednisone (dose range 5-20mg), four used hydroxychloroquine (200mg BID), two used Mycophenylate (720 bid to 720 tid), and one used tacrolimus (4 bid).
(TIF)

## References

[1] Kemler R. From cadherins to catenins: cytoplasmic protein interactions and regulation of cell adhesion. Trends Genet. 1993;9(9):317–21. 8236461 10.1016/0168-9525(93)90250-l

[2] McCREA PD, Turck CW, Gumbiner B. A homolog of the armadillo protein in Drosophila (plakoglobin) associated with E-cadherin. Science. 1991;254(5036):1359–61. 1962194 10.1126/science.1962194

[3] Behrens J, von Kries JP, Kühl M, Bruhn L, Wedlich D, Grosschedl R, et al Functional interaction of β-catenin with the transcription factor LEF-1. Nature. 1996;382(6592):638–42. 8757136 10.1038/382638a0

[4] Ikeda S, Kishida S, Yamamoto H, Murai H, Koyama S, Kikuchi A. Axin, a negative regulator of the Wnt signaling pathway, forms a complex with GSK-3β and β-catenin and promotes GSK-3β-dependent phosphorylation of β-catenin. The EMBO Journal. 1998;17(5):1371–84. 9482734 10.1093/emboj/17.5.1371 PMC1170485

[5] MacDonald BT, Tamai K, He X. Wnt/β-catenin signaling: components, mechanisms, and diseases. Dev Cell. 2009;17(1):9–26. 10.1016/j.devcel.2009.06.016 19619488 PMC2861485

[6] Shtutman M, Zhurinsky J, Simcha I, Albanese C, D’Amico M, Pestell R, et al The cyclin D1 gene is a target of the β-catenin/LEF-1 pathway. Proc Natl Acad Sci U S A. 1999;96(10):5522–7. 10318916 10.1073/pnas.96.10.5522 PMC21892

[7] Pazdera TM, Janardhan P, Minden JS. Patterned epidermal cell death in wild-type and segment polarity mutant Drosophila embryos. Development. 1998;125(17):3427–36. 9693146 10.1242/dev.125.17.3427

[8] Grigoryan T, Wend P, Klaus A, Birchmeier W. Deciphering the function of canonical Wnt signals in development and disease: conditional loss-and gain-of-function mutations of β-catenin in mice. Genes Dev. 2008;22(17):2308–41. 10.1101/gad.1686208 18765787 PMC2749675

[9] Nishisho I, Nakamura Y, Miyoshi Y, Miki Y, Ando H, Horii A, et al Mutations of chromosome 5q21 genes in FAP and colorectal cancer patients. Science. 1991;253(5020):665–9. 1651563 10.1126/science.1651563

[10] Laken SJ, Petersen GM, Gruber SB, Oddoux C, Ostrer H, Giardiello FM, et al Familial colorectal cancer in Ashkenazim due to a hypermutable tract in APC. Nat Genet. 1997;17(1):79–83. 9288102 10.1038/ng0997-79

[11] Barker N, Clevers H. Mining the Wnt pathway for cancer therapeutics. Nature Reviews Drug Discovery. 2006;5(12):997–1014. 17139285 10.1038/nrd2154

[12] Rivera MN, Kim WJ, Wells J, Driscoll DR, Brannigan BW, Han M, et al An X chromosome gene, WTX, is commonly inactivated in Wilms tumor. Science. 2007;315(5812):642–5. 17204608 10.1126/science.1137509

[13] Satoh S, Daigo Y, Furukawa Y, Kato T, Miwa N, Nishiwaki T, et al AXIN1 mutations in hepatocellular carcinomas, and growth suppression in cancer cells by virus-mediated transfer of AXIN1. Nat Genet. 2000;24(3):245–50. 10700176 10.1038/73448

[14] Reya T, Duncan AW, Ailles L, Domen J, Scherer DC, Willert K, et al A role for Wnt signalling in self-renewal of haematopoietic stem cells. Nature. 2003;423(6938):409–14. 12717450 10.1038/nature01593

[15] Staal FJ, Meeldijk J, Moerer P, Jay P, van de Weerdt B, Vainio S, et al Wnt signaling is required for thymocyte development and activates Tcf‐1 mediated transcription. Eur J Immunol. 2001;31(1):285–93. 11265645 10.1002/1521-4141(200101)31:1<285::AID-IMMU285>3.0.CO;2-D

[16] Ranheim EA, Kwan HC, Reya T, Wang Y-K, Weissman IL, Francke U. Frizzled 9 knock-out mice have abnormal B-cell development. Blood. 2005;105(6):2487–94. 15572594 10.1182/blood-2004-06-2334

[17] Yang P, An H, Liu X, Wen M, Zheng Y, Rui Y, et al The cytosolic nucleic acid sensor LRRFIP1 mediates the production of type I interferon via a [beta]-catenin-dependent pathway. Nat Immunol. 2010;11(6):487–94. 10.1038/ni.1876 20453844

[18] Kim JH, Kim B, Cai L, Choi HJ, Ohgi KA, Tran C, et al Transcriptional regulation of a metastasis suppressor gene by Tip60 and β-catenin complexes. Nature. 2005;434(7035):921–6. 15829968 10.1038/nature03452

[19] Deng J, Miller SA, Wang H-Y, Xia W, Wen Y, Zhou BP, et al β-catenin interacts with and inhibits NF-κB in human colon and breast cancer. Cancer Cell. 2002;2(4):323–34. 10.1016/S1535-6108(02)00154-X. 12398896

[20] Manicassamy S, Reizis B, Ravindran R, Nakaya H, Salazar-Gonzalez RM, Wang Y-c, et al Activation of β-Catenin in Dendritic Cells Regulates Immunity Versus Tolerance in the Intestine. Science. 2010;329(5993):849–53. 10.1126/science.1188510 20705860 PMC3732486

[21] Jiang A, Bloom O, Ono S, Cui W, Unternaehrer J, Jiang S, et al Disruption of E-cadherin-mediated adhesion induces a functionally distinct pathway of dendritic cell maturation. Immunity. 2007;27(4):610–24. 17936032 10.1016/j.immuni.2007.08.015 PMC2151979

[22] Diarra D, Stolina M, Polzer K, Zwerina J, Ominsky MS, Dwyer D, et al Dickkopf-1 is a master regulator of joint remodeling. Nat Med. 2007;13(2):156–63. http://www.nature.com/nm/journal/v13/n2/suppinfo/nm1538_S1.html. 17237793 10.1038/nm1538

[23] Yu Q, Sharma A, Ghosh A, Sen JM. T Cell Factor-1 Negatively Regulates Expression of IL-17 Family of Cytokines and Protects Mice from Experimental Autoimmune Encephalomyelitis. The Journal of Immunology. 2011;186(7):3946–52. 10.4049/jimmunol.1003497 21339363 PMC3158594

[24] Tveita AA, Rekvig OP. Alterations in Wnt pathway activity in mouse serum and kidneys during lupus development. Arthritis Rheum. 2011;63(2):513–22. 10.1002/art.30116 21280006

[25] Brault V, Moore R, Kutsch S, Ishibashi M, Rowitch DH, McMahon AP, et al Inactivation of the (β)-catenin gene by Wnt1-Cre-mediated deletion results in dramatic brain malformation and failure of craniofacial development. Development. 2001;128(8):1253–64. 11262227 10.1242/dev.128.8.1253

[26] Clausen B, Burkhardt C, Reith W, Renkawitz R, Förster I. Conditional gene targeting in macrophages and granulocytes using LysMcre mice. Transgenic Res. 1999;8(4):265–77. 10621974 10.1023/a:1008942828960

[27] Morel L, Blenman KR, Croker BP, Wakeland EK. The major murine systemic lupus erythematosus susceptibility locus, Sle1, is a cluster of functionally related genes. Proc Natl Acad Sci U S A. 2001;98(4):1787–92. 11172029 10.1073/pnas.031336098 PMC29335

[28] Morel L, Croker BP, Blenman KR, Mohan C, Huang G, Gilkeson G, et al Genetic reconstitution of systemic lupus erythematosus immunopathology with polycongenic murine strains. Proc Natl Acad Sci U S A. 2000;97(12):6670–5. 10.1073/pnas.97.12.6670 10841565 PMC18697

[29] Subramanian S, Tus K, Li Q-Z, Wang A, Tian X-H, Zhou J, et al A Tlr7 translocation accelerates systemic autoimmunity in murine lupus. Proc Natl Acad Sci U S A. 2006;103(26):9970–5. 16777955 10.1073/pnas.0603912103 PMC1502563

[30] Helyer BJ, Howie JB. Renal disease associated with positive lupus erythematosus tests in a cross-bred strain of mice. Nature. 1963;197:197 Epub 1963/01/12. . 13953664 10.1038/197197a0

[31] Orme J, Mohan C. Macrophages and neutrophils in SLE—An online molecular catalog. Autoimmun Rev. 2012;11(5):365–72. 10.1016/j.autrev.2011.10.010 22036828

[32] Liu J, Beller DI. Distinct pathways for NF-κB regulation are associated with aberrant macrophage IL-12 production in lupus-and diabetes-prone mouse strains. The Journal of Immunology. 2003;170(9):4489–96. 12707325 10.4049/jimmunol.170.9.4489

[33] Wakui M, Kim J, Butfiloski EJ, Morel L, Sobel ES. Genetic Dissection of Lupus Pathogenesis: Sle3/5 Impacts IgH CDR3 Sequences, Somatic Mutations, and Receptor Editing. The Journal of Immunology. 2004;173(12):7368–76. 15585861 10.4049/jimmunol.173.12.7368

[34] Abbas AR, Wolslegel K, Seshasayee D, Modrusan Z, Clark HF. Deconvolution of blood microarray data identifies cellular activation patterns in systemic lupus erythematosus. PLoS ONE. 2009;4(7):e6098 10.1371/journal.pone.0006098 19568420 PMC2699551

[35] Wang X-d, Huang X-f, Yan Q-r. Aberrant activation of the WNT/β-catenin signaling pathway in lupus nephritis. PLoS ONE. 2014;9(1):e84852 10.1371/journal.pone.0084852 24465439 PMC3897389

[36] Enzler T, Bonizzi G, Silverman GJ, Otero DC, Widhopf GF, Anzelon-Mills A, et al Alternative and classical NF-κB signaling retain autoreactive B cells in the splenic marginal zone and result in lupus-like disease. Immunity. 2006;25(3):403–15. 16973390 10.1016/j.immuni.2006.07.010

[37] Huang Z, Wang R, Xie H, Shang W, Manicassamy S, Sun Z. Stabilized β-catenin potentiates Fas-mediated T cell apoptosis. The Journal of Immunology. 2008;180(10):6586–92. 18453577 10.4049/jimmunol.180.10.6586

[38] Ding Y, Shen S, Lino AC, de Lafaille MAC, Lafaille JJ. Beta-catenin stabilization extends regulatory T cell survival and induces anergy in nonregulatory T cells. Nat Med. 2008;14(2):162–9. 10.1038/nm1707 18246080

[39] Pascual V, Banchereau J, Palucka A. The central role of dendritic cells and interferon-alpha in SLE. Curr Opin Rheumatol. 2003;15(5):548–56. . 12960479 10.1097/00002281-200309000-00005

[40] Thompson MD, Dar MJ, Monga SP. Pegylated interferon alpha targets Wnt signaling by inducing nuclear export of β-catenin. J Hepatol. 2011;54(3):506–12. 10.1016/j.jhep.2010.07.020 21093092 PMC3052972

[41] Ozawa M, Ringwald M, Kemler R. Uvomorulin-catenin complex formation is regulated by a specific domain in the cytoplasmic region of the cell adhesion molecule. Proc Natl Acad Sci U S A. 1990;87(11):4246–50. 2349235 10.1073/pnas.87.11.4246 PMC54085

[42] Nelson WJ, Nusse R. Convergence of Wnt, ß-Catenin, and Cadherin Pathways. Science. 2004;303(5663):1483–7. 10.1126/science.1094291 15001769 PMC3372896

