# Supplementary figures and images for: Leukocyte Beta-Catenin Expression Is Disturbed in Systemic Lupus Erythematosus
Source: PLoS One. 2016 Aug 22;11(8):e0161682. doi: 10.1371/journal.pone.0161682 (PMC4993388; doi:10.1371/journal.pone.0161682)

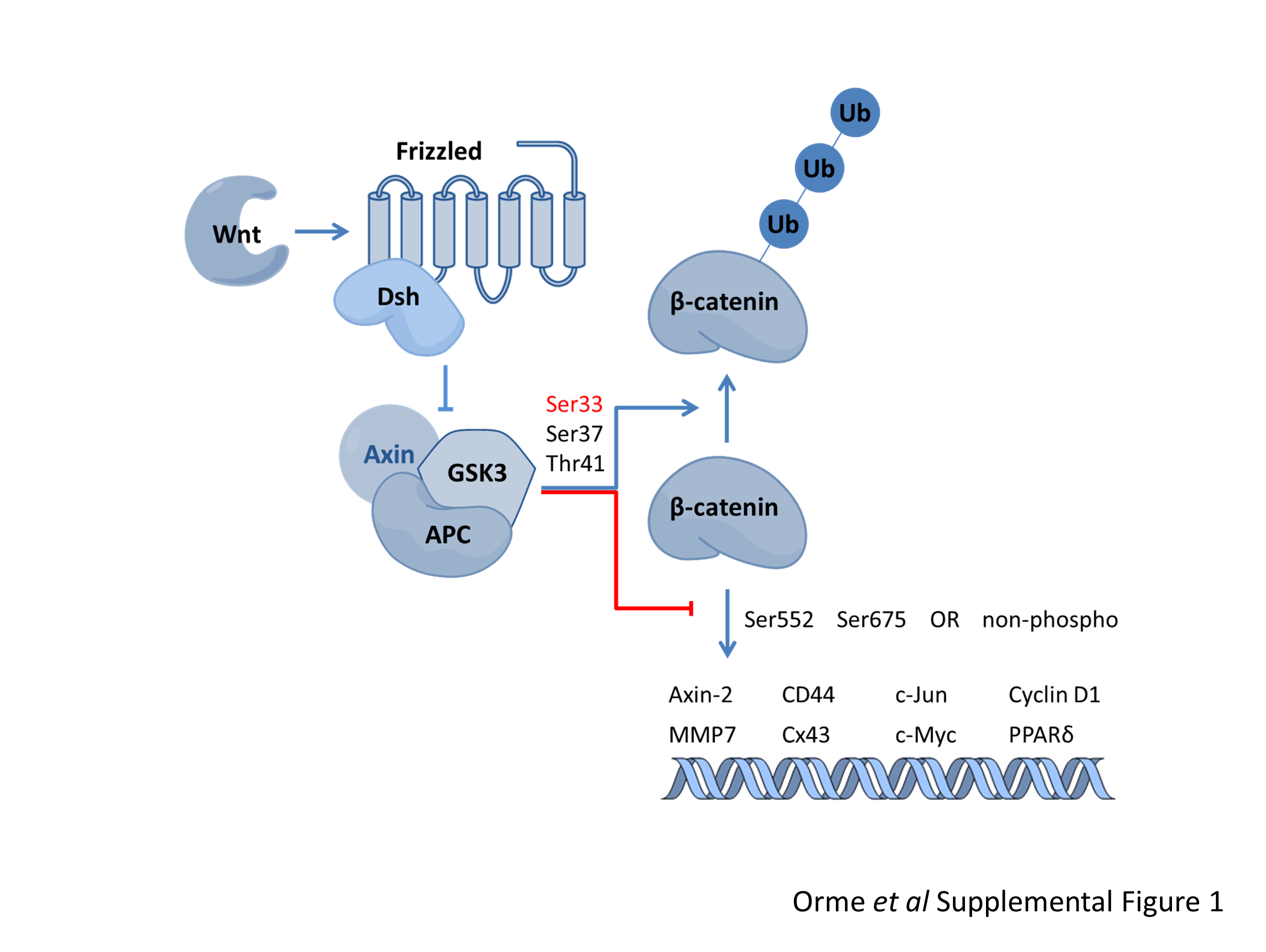

Supplement: S1 Fig — (TIF) [file pone.0161682.s001.TIF]

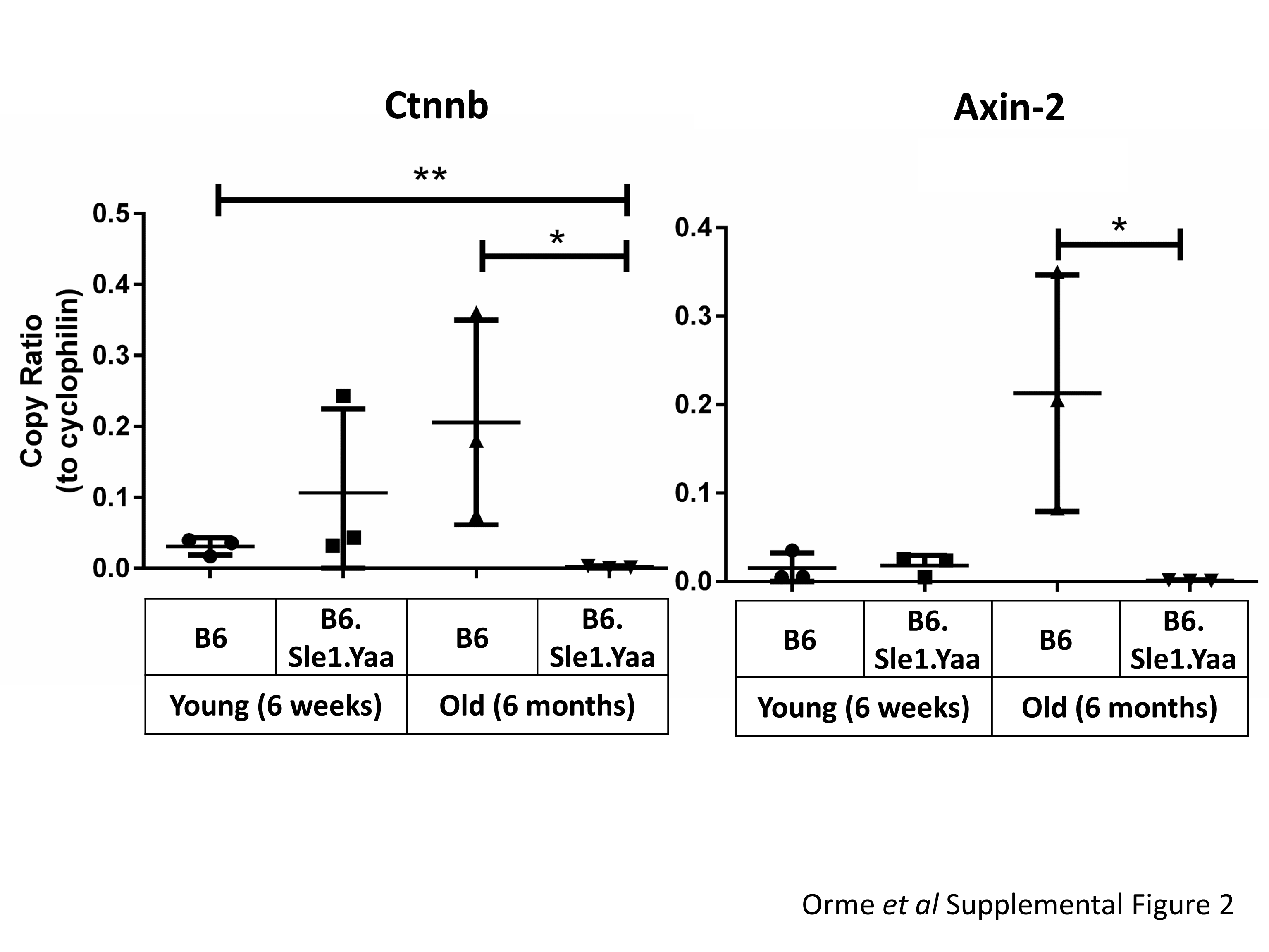

Supplement: S2 Fig — Splenocytes from age-matched young (6 week) and old (6 month) healthy control B6 and lupus-prone B6.Sle1.Yaa mice were isolated and tested for Ctnnb and Axin2 expression by RT-PCR. Older lupus-prone mice exhibited significantly lower Ctnnb and Axin2 transcripts than healthy age-matched controls (p = 0.0354, p = 0.0259). In contrast, young lupus-prone mice expressed Ctnnb and Axin2 transcripts at levels similar to those in young B6 controls. (TIF) [file pone.0161682.s002.TIF]

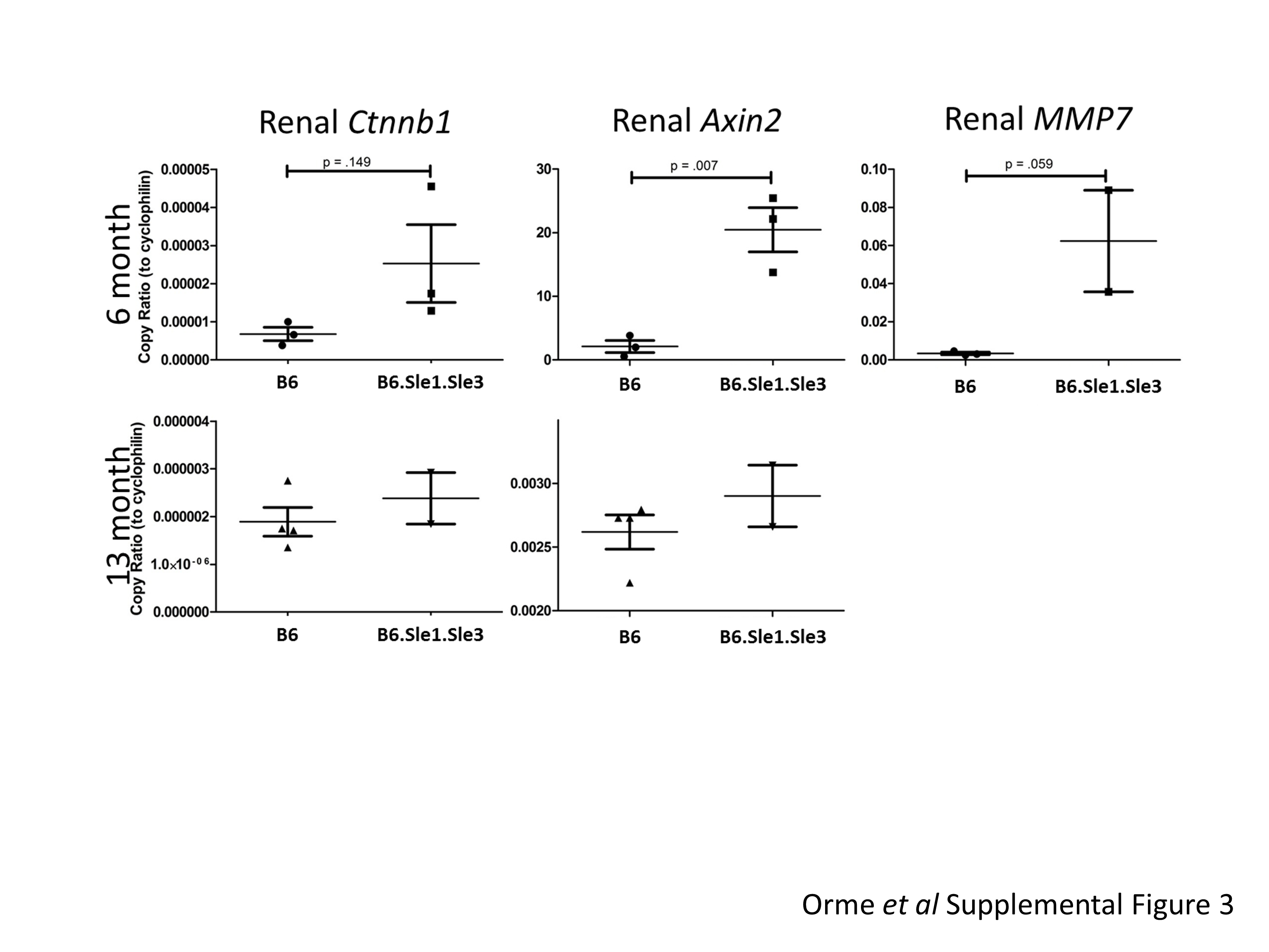

Supplement: S3 Fig — Beta catenin expression and that of its transcriptional targets Axin2 and MMP7 were compared by RT-PCR in 4 month-old B6 healthy control and B6.Sle1.Sle3 lupus-prone kidneys. Transcription of these genes is elevated in B6.Sle1.Sle3 lupus-prone mice. (TIF) [file pone.0161682.s003.TIF]

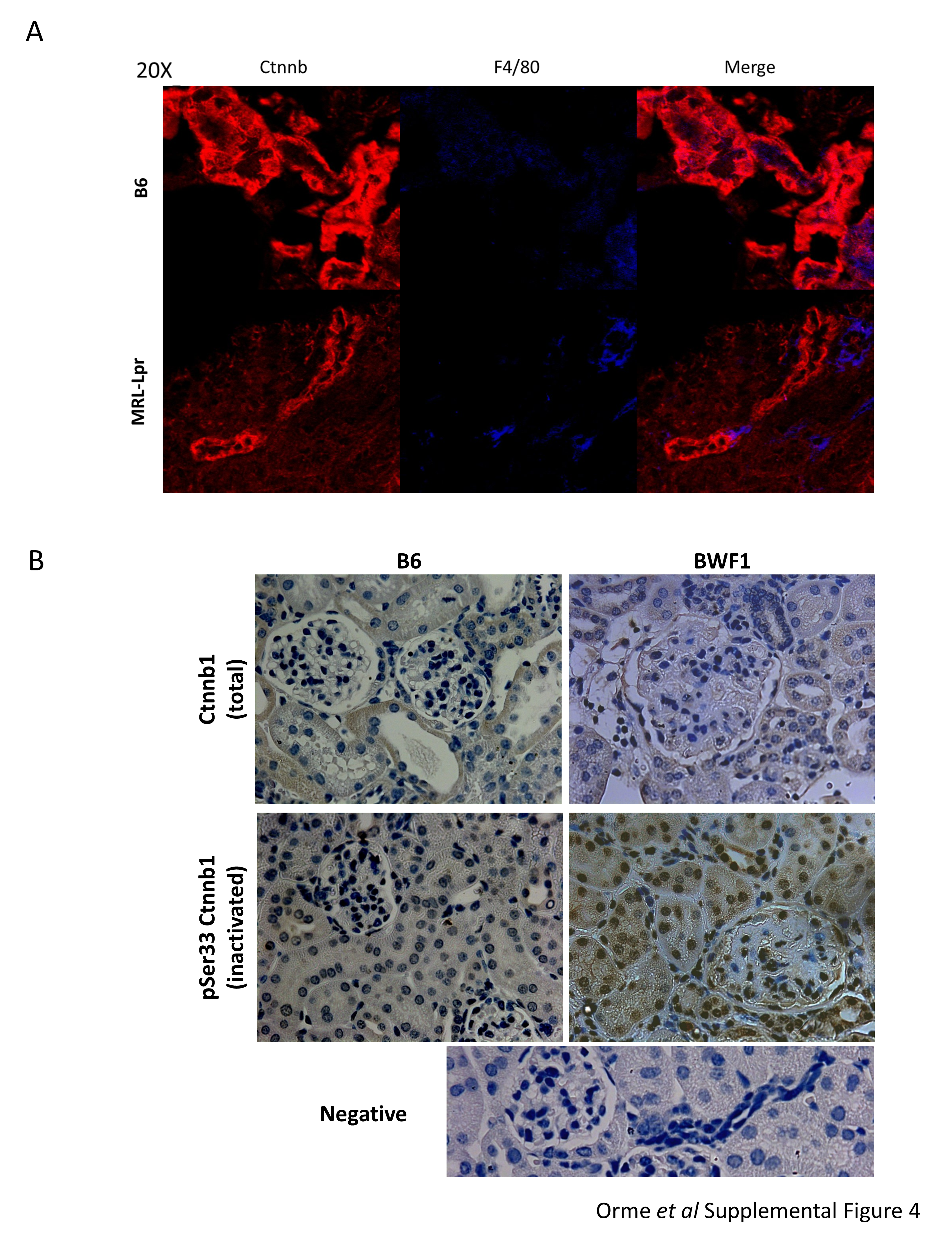

Supplement: S4 Fig — (A) Kidneys were isolated, prepared, and stained for β-catenin (red) and macrophage marker F4/80 (blue). (B) Kidneys were isolated, prepared, and stained for total β-catenin (top) and inactivated/structural pSer33 β-catenin (bottom). (TIF) [file pone.0161682.s004.tif]

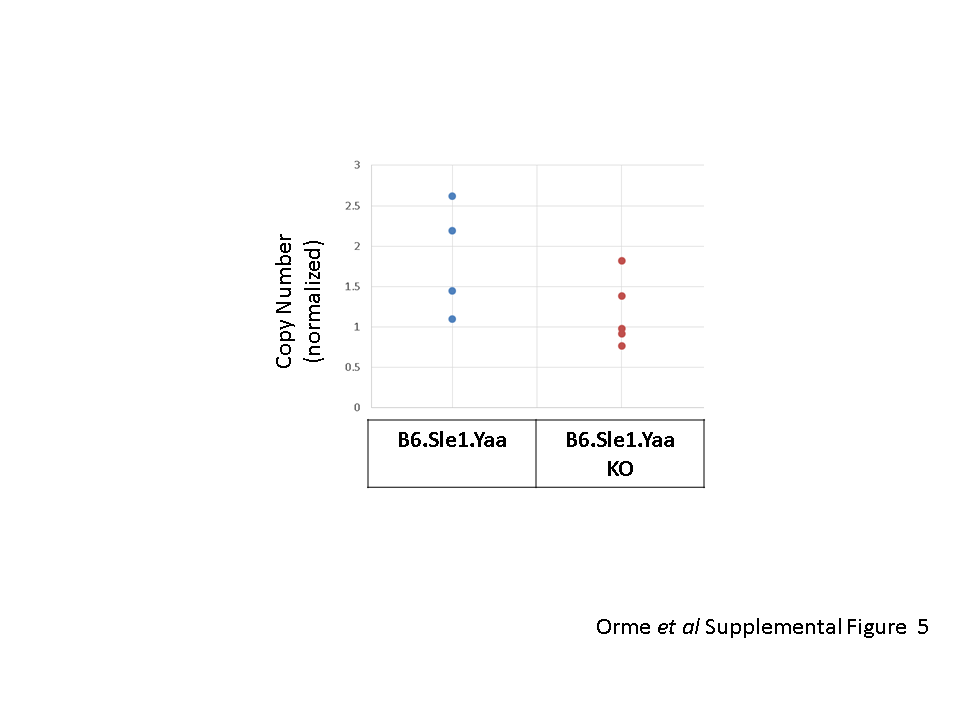

Supplement: S5 Fig — Peripheral blood was taken from young (2–4 wk) B6.Sle1.Yaa.Ctnnbfl/fl and B6.Sle1.Yaa.LyzM-cre mice, RNA was isolated, and RT-PCR was performed to determine gross levels of leukocyte Ctnnb in each mouse. Due to animal size, there was insufficient blood extracted for a subset of samples to perform RT-PCR. (TIF) [file pone.0161682.s005.tif]

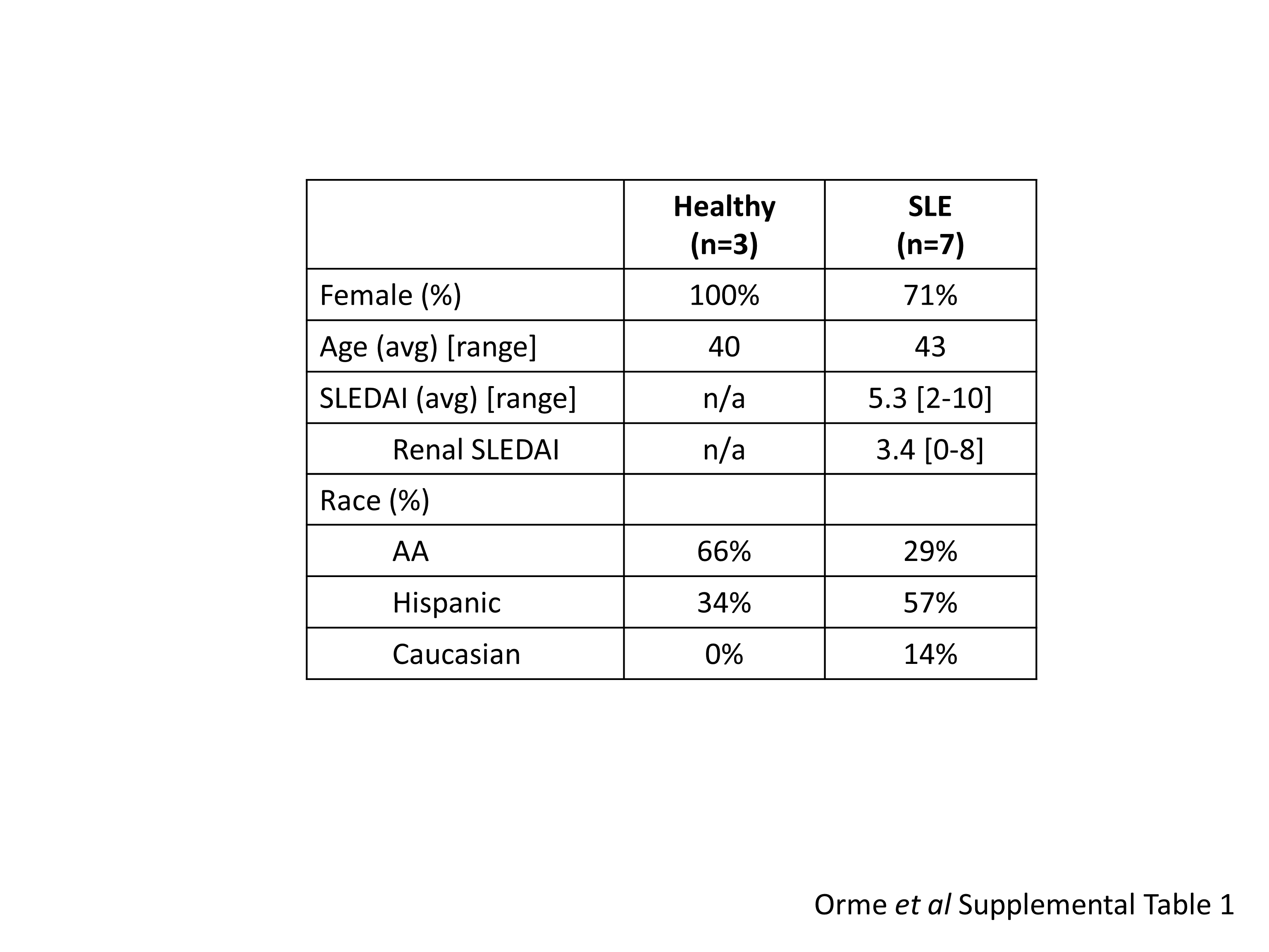

Supplement: S1 Table — Gender, race, age, and SLEDAI scores are given for healthy controls and SLE patients from whom samples were obtained for Fig 1. Of SLE patients, four used Prednisone (dose range 5-20mg), four used hydroxychloroquine (200mg BID), two used Mycophenylate (720 bid to 720 tid), and one used tacrolimus (4 bid). (TIF) [file pone.0161682.s006.TIF]
